# Association of neovascular age-related macular degeneration with migraine

**DOI:** 10.1038/s41598-022-05638-5

**Published:** 2022-02-02

**Authors:** Tung-Mei Kuang, Sudha Xirasagar, Yi-Wei Kao, Jau-Der Ho, Herng-Ching Lin

**Affiliations:** 1 Department of Ophthalmology, Taipei Veterans General Hospital, Taipei, Taiwan; 2 School of Medicine, National Yang-Ming University, Taipei, Taiwan; 3 Research Center of Sleep Medicine, College of Medicine, Taipei Medical University, Taipei, Taiwan; 4 Department of Health Services Policy and Management, Arnold School of Public Health, University of South Carolina, Columbia, USA; 5 Big Data Research Center, Taipei Medical University, Taipei, Taiwan; 6 Graduate Institute of Business Administration, College of Management, Fu Jen Catholic University, New Taipei City, Taiwan; 7 Department of Ophthalmology, Taipei Medical University Hospital, Taipei, Taiwan; 8 Sleep Research Center, Taipei Medical University Hospital, Taipei, Taiwan; 9 School of Health Care Administration, College of Management, Taipei Medical University, 250 Wu-Hsing Street, Taipei, 110 Taiwan

**Keywords:** Neurology, Risk factors

## Abstract

Patients with early onset vascular pathology have been reported to manifest neovascular age-related macular degeneration (AMD). While the blood vessels involved in pathogenesis of migraine remains controversial, it is generally accepted that a major contributor is blood vessel pathology. This study aimed to examine the association between migraine and AMD using a nationwide population-based dataset. Retrospective claims data were collected from the Taiwan National Health Insurance Research Database. We identified 20,333 patients diagnosed with neovascular AMD (cases), and we selected 81,332 propensity score-matched controls from the remaining beneficiaries in Taiwan’s National Health Insurance system. We used Chi-square tests to explore differences in the prevalence of migraine prior to the index date between cases and controls. We performed multiple logistic regressions to estimate the odds of prior migraine among neovascular AMD patients vs. controls after adjusting for age, sex, monthly income, geographic location, residential urbanization level, hyperlipidemia, diabetes, coronary heart disease, hypertension, and previous cataract surgery. A total of 5184 of sample patients (5.1%) had a migraine claim before the index date; 1215 (6.1%) among cases and 3969 (4.9%) among controls (*p* < 0.001), with an unadjusted OR of 1.239 (95% CI 1.160~1.324, *p* < 0.001) for prior migraine among cases relative to controls. Furthermore, the adjusted OR was 1.201 (95% CI 1.123~1.284; *p* < 0.001) for AMD cases relative to controls. The study offers population-based evidence that persons with migraine have 20% higher risk of subsequently being diagnosed with neovascular AMD.

## Introduction

Age-related macular degeneration (AMD) is one of the leading causes of irreversible blindness and visual impairment worldwide^1–4^. A recent meta-analysis reported that AMD was responsible for 8.7% of all global blindness, with the proportion of late-stage AMD being 0.4%. While AMD can arise among middle-aged individuals, studies show that the prevalence is non-linear and steeply rises after 75 years of age among all ethnicities^1^.

Traditionally, neovascular AMD has been considered as a disease confined to the eye. In recent years, research has accumulated on the co-occurrence of diseases affecting the eyes, heart and brain due to similar underlying vascular pathology, particularly showing that endothelial dysfunction may play a role in many human diseases^5^. Patients with early onset vascular pathology have been reported to manifest AMD and other allied cardiovascular and cerebrovascular diseases later in life^6^. For example, AMD was associated with a 1.58-fold increased risk of heart failure after adjustment for potential confounders in a population-based nested case–control study. This significant association was evident in both nonexudative and exudative AMD subgroups^7^. The relationship between AMD and cardiovascular disease is supported by genetic studies that the pleiotropic 15q24.1 association signal may have a shared mechanism between blood pressure regulation and choroidal neovascularization with a potential involvement of CYP1A1^8^. Further genetic evidence from a total of 33,526 individuals predominantly of European ancestry from the International Age-related Macular Degeneration Genomics Consortium showed that increasing HDL-cholesterol (particularly via CETP inhibition) is a causal risk factor for AMD and that increasing HDL-cholesterol will increase AMD risk^9^. Migraine is the third most common medical condition worldwide and is the most common neurological disorder^10^. While the blood vessels involved in its pathogenesis remains controversial—cerebral or meningeal vessel vasodilation, it is generally accepted that a major contributor is blood vessel pathology, specifically of the endothelial cells. A recent genome-wide analysis of 102,084 migraine cases showed that genomic annotations among migraine-associated variants were enriched in both vascular and central nervous system tissue/cell types supporting that neurovascular mechanism underlies its pathophysiology^11^. On the other hand, there is evidence that two variants in ARMS2/HTRA1 were associated with increased risk of early AMD as well as for late AMD^12^, and selected genetic scores showed a significant correlation between AMD and migraine^13^. With current evidence showing AMD to be a comorbidity of cardiovascular and neurodegenerative diseases^5,14–17^ like dementia^18^ and Alzheimer’s disease^16^, we investigated whether migraine is associated with age-related neovascular AMD using a nationwide population-based dataset.

## Methods

### Database

We retrieved claims data on sample patients from the Taiwan National Health Insurance (NHI) Research Database (NHIRD). The NHIRD includes medical claims data and beneficiary registry files for about 1 million beneficiaries who represent a stratified random sample of approximately 99% of Taiwan’s population (about 24.02 million as of December 2019) in Taiwan’s NHI program. Many scientists in Taiwan have used the NHIRD to carry out longitudinal studies of diseases and treatments using claims data on follow-up medical services.

The study was approved by the institutional review board of Taipei Medical University (TMU-JIRB N202004019). This study adhered to the Declaration of Helsinki. This study used administrative dataset so we did not need patient informed consent.

### Identification of cases and controls

We identified 26,209 patients from the NHIRD with a first-time diagnosis of neovascular AMD (ICD-9-CM code 362.52 or ICD-10-CM code H35.32) during an ambulatory care visit between January 2010 and December 2016. To address the concern about diagnostic validity in administrative datasets, we included only patients with a diagnosis of neovascular AMD in at least two claims filed by ophthalmologists during the sample selection period (*n* = 21,206). We assigned the first date of the neovascular AMD diagnosis as the index date. We excluded 873 patients under 40 years of age because neovascular AMD is rare in this age group. The remaining 20,333 patients with neovascular AMD were included as cases in the study.

We selected matched controls from the remaining NHIRD beneficiaries from the Registry. Controls were identified at the rate of four propensity score-matched controls per case (*n* = 81,332). We first calculated a propensity score for each enrollee based on patient demographics (age, sex, monthly income category, geographic location, and urbanization level of the patient’s residence), and medical comorbidities known to be associated with AMD risk, hyperlipidemia, diabetes, coronary heart disease, and hypertension if they were present before the index date. We may be unable to find an exact matching propensity score to match controls. Therefore, we used the method of nearest neighbor within calipers to match controls (apriori value for the calipers is ± 0.01). In addition, controls were matched to a given neovascular AMD patient if they had utilized any ambulatory care service in the index year of the neovascular AMD case. We defined the date of control patients’ first ambulatory care visit during the index year of their matched case as their index date. We also assured that all controls had shown evidence of eye examination by an ophthalmologist within 2 years prior to the index date in order to avoid the possibility of undiagnosed AMD. The final study sample consisted of 20,333 cases and 81,332 controls.

We estimated the odds of a previous diagnosis of migraine prior to the index date for cases relative to controls by matching the study patients to ambulatory care claims prior to the index date. We identified study patients with a prior migraine diagnosis based on ICD-9-CM code 346 or ICD-10-CM code G43 found at least in two medical care claims within 3 years prior to the index date to enhance diagnostic validity.

### Statistical analysis

We used the SAS system (SAS System for Windows, V, 8.2, SAS Institute, Cary, NC) for statistical analyses. We used Chi-square tests to study differences in demographics and medical comorbidities between cases and controls. We accounted for these factors and whether the patient had cataract surgery prior to the index date in the adjusted analysis. Logistic regression analysis was used to examine the association of neovascular AMD with previously diagnosed migraine. The conventional *p* ≤ 0.05 was used to assess statistical significance.

## Results

Of 101,665 study patients, the mean age was 71.4 years, 71.3 and 71.5 years among cases and controls, respectively (*p* = 0.015). Table 1 shows that small but statistically significant differences between cases and controls on sex (*p* = 0.023), monthly income (*p* = 0.021), geographical location (*p* = 0.004), and residential urbanization level (*p* = 0.002). Cases and controls also significantly differed on the prevalence of hypertension (68.7% vs. 69.7%, *p* = 0.004), coronary heart disease (31.9% vs. 29.4%, *p* < 0.001), and cataract surgery preceding the index date (21.7% vs. 13.2%, *p* < 0.001). Table 1 also shows the prevalence of prior migraine among cases and controls. A total of 5,184 sample patients (5.1%) had migraine before the index date; 1,215 cases (6.1%) and 3969 controls (4.9%); the difference was statistically significant, *p* < 0.001.

**Table 1:** Demographic characteristics of persons with neovascular age-related macular degeneration (AMD) and controls in Taiwan (n = 101,665).

Variable	Patients with neovascular AMD (*n* = 20,333)		Controls (*n* = 81,332)		*p* value
	Total no	%	Total no	%	
Presence of migraine	1215	6.1	3969	4.9	< 0.001
Age, mean (SD)	71.3 (11.4)	71.5 (12.0)	0.015		
Males	12,277	60.4	48,396	59.5	0.023
**Monthly Income**					0.021
< NT$1~15,841	7281	35.8	28,335	34.8	
NT$15,841~25,000	7512	36.9	30,764	37.8	
≥ NT$25,001	5540	27.2	22,233	27.3	
**Geographic region**					0.004
Northern	10,839	53.3	42,614	52.4	
Central	4658	22.9	19,030	23.4	
Eastern	481	2.4	1706	2.1	
Southern	4355	21.4	17,982	22.1	
**Urbanization level**					0.002
1 (most urbanized)	8442	41.5	33,531	41.2	
2	7407	36.4	29,959	36.8	
3	3210	15.8	13,299	16.4	
4	569	2.8	2002	2.5	
5 (least urbanized)	705	3.5	2541	3.1	
Diabetes	7807	38.4	30,728	37.8	0.108
Hypertension	13,967	68.7	56,705	69.7	0.004
Coronary heart disease	6494	31.9	23,940	29.4	< 0.001
Prior cataract surgery	4422	21.7	10,765	13.2	< 0.001
Hyperlipidemia	9296	45.7	36,639	45	0.087

Table 2 presents the adjusted OR of prior migraine, 1.201 (95% CI 1.123~1.284; *p* < 0.001) after adjusting for age, sex, monthly income, geographic location, urbanization, hyperlipidemia, diabetes, coronary heart disease, hypertension, and cataract surgery.

**Table 2:** Covariate-adjusted odds of prior migraine (OR and 95% confidence interval, CIs) among neovascular age-related macular degeneration vs. controls (n = 101,665).

Variable	Presence of neovascular age-related macular degeneration		
	Adjusted OR	95% CI	*p* value
Prior migraine	1.201	1.123~1.284	< 0.001
Age	0.993	0.991~0.994	< 0.001
**Monthly income**			
< NT$15,841 (reference group)	1.000		
NT$15,841~25,000	0.928	0.892~0.966	< 0.001
≥ NT$25,001	0.945	0.906~0.984	0.007
**Geographic region**			
Northern (reference group)	1.000		
Central	0.957	0.918~0.999	0.045
Eastern	0.865	0.687~1.089	0.217
Southern	0.91	0.871~0.952	< 0.001
**Urbanization level**			
1 (reference group)	1.000		
2	1.017	0.980~1.056	0.375
3	1.017	0.964~1.074	0.535
4	1.274	1.029~1.578	0.026
5	1.163	1.059~1.277	0.002
Hyperlipidemia	1.002	0.968~1.036	0.927
Diabetes	1.012	0.978~1.047	0.500
Hypertension	0.911	0.878~0.945	< 0.001
Coronary heart disease	1.125	1.087~1.165	< 0.001
Prior cataract surgery	1.935	1.857~2.015	< 0.001

## Discussion

Our results show 20% higher risk of neovascular AMD among migraine patients than control patients without migraine. To our knowledge, there has been no report of an association between migraine and subsequent neovascular AMD.

Neovascular AMD is characterized by the formation of choroidal neovascular fibrovascular complexes that are generated from the chorio-capillaries through defective Bruch’s membrane. The pathogenesis is not fully understood, but vascular endothelial growth factors (VEGF) play an important role in its development^19^. VEGF-A promotes vascular endothelial cell proliferation and division, as well as neovascularization and supporting the new vessels to survive. VEGF-A is an inflammatory, cellular chemotactic factor^177^ and increases vascular permeability^21^. Choroidal endothelial cells proliferate and form new vessels and also secrete angiogenic and inflammatory cytokines as well as growth factors^20^.

The major contributing pathophysiological event to initiate migraine was cerebral and meningeal arteries vasodilation. Recently, it is debatable at what time point vasodilation may play a role and some even raised the question whether vasodilation is necessary to incite a migraine episode.^10^ Despite these controversies, vessels are still an important potential contributors to migraine development. Endothelial cells of blood vessels mediate immune cell recruitment and downstream inflammatory signaling pathways. They also express a variety of channels and receptors thought to be involved in the detection of noxious stimuli, and along with neurons, endothelial cells may potentiate the responses to noxious stimuli. For example, c-type natriuretic peptide may represent an endothelial derived molecule that is capable of triggering attacks. Other factors like endothelin-1 (ET-1), a potent vasodilator and mediator elevated in human plasma at the onset of migraine attacks, sensitizes nociceptors to mechanical-stimuli via endothelial cell-mediated release of adenosine triphosphate leading to hyperalgesia.

Recent epidemiological studies have shown that age-related macular degeneration is associated with stroke, cardiovascular disease and Alzheimer’s disease^5,14–17^. In the Atherosclerosis Risk in Communities Study, 576 participants were diagnosed with early and 15 late AMD. An increased risk of stroke was noted, with a stronger association observed for intracerebral hemorrhage than cerebral infarction was observed for all AMD over 13-year follow-up^17^. The same study also showed that participants with signs of late-stage AMD were more likely to have a coronary heart disease event over 10-year follow-up (10-year incidence 30.9% vs 10.0% among those without late-stage AMD). Further, late-stage AMD was significantly associated with mortality (10-year cumulative mortality rate 23.5% vs 8.9%)^22^. Similar to this study, the Blue Mountains Eye Study also noted that late-stage AMD predicted five-fold higher cardiovascular mortality and 10-fold higher stroke mortality after adjusting for age and sex^23^.

Increasing evidence supports that AMD, cardiovascular disease and stroke share common risk factors and pathological mechanisms^23^. It has been proposed that inflammatory markers in the eye are linked to and co-occur with the activation of inflammatory pathways in the heart, and that endothelial dysfunction and oxidative stress are common in AMD and cardiovascular disease^23,24^. On another note, individuals with AMD are 50% more likely to develop Alzheimer’s disease. The two diseases share several degenerative and pathological features such as oxidative stress, inflammation, and deposition of amyloid-rich materials^25,26^. Microvascular abnormalities in early Alzheimer’s disease show similarities to the patterns of vascular dysfunction found in neovascular AMD^27^.

Similarly, dysregulation of vascular endothelial growth factors is observed at the onset of migraine attacks^28–31^. Endothelial cell-mediated release of endothelin-1, a potent vasodilator and migraine mediator are elevated in human plasma at the onset of migraine attacks^29^. It sensitizes nociceptors to mechanical-stimuli via endothelial cell-mediated release of ATP leading to migraine^30,31^. In addition, c-type natriuretic peptide which is secreted by endothelium is observed to induce thermal hyperalgesia in mice^32^. Endothelial cells recruit immune cells and inflammatory signaling pathways which are thought to be crucial in the pathogenesis of migraine. Endothelial cells also express a variety of channels and receptors that are involved in the detection of noxious stimuli during a migraine attack^10^. These pathways may overlap with those found in neovascular AMD. The common molecular mechanisms underlying neovascular AMD, cardiovascular disease and migraine remain intriguing and complex, worthy of further investigation.

This case-control study has some unique strengths. First, the NHIRD is a nationwide population-based database of a large representative sample of the entire Taiwanese population of over 23 million, preempting the selection bias typical of observational studies of clinical populations. Further with more than 95% of the Taiwanese population being of Han Chinese ethnicity, potential bias due to ethnic composition is avoided. Further, although there were very small but statistically significant differences on the matching variables, the statistical significance largely attributable to large sample size. The magnitudes of difference support validity of the propensity score matching process.

There as some study limitations. The NHIRD lacks data on family history and lifestyle risk factors for neovascular AMD such as, dietary habits, smoking, alcohol consumption, and genetic factors. Particularly, smoking is documented to be highly associated with AMD. One study found that current smokers had a 6.6-fold increased risk of neovascular AMD vs those who had never smoked in subjects younger than 85 years^33^. Further survey studies are needed to examine the association between migraine and AMD by taking smoking into consideration. Second, the NHIRD did not provide the findings or images generated from ophthalmologic evaluations (including dilated eye examinations, fluorescein dye retinal angiography, optical coherence tomography, etc.) which provide details of the severity of AMD. Severity of neovascular AMD and migraine cannot be ascertained from the ICD-9-CM diagnosis code. Third, some patients with neovascular AMD may not seek medical care due to a lack of awareness of visual impairment or of the role of AMD in their visual loss. Therefore, potential misclassification bias remains a limitation as some controls may have undiagnosed neovascular AMD. However, such bias would likely drive the result towards the null hypothesis, indicating that our detected difference may be an underestimation of the actual difference between the groups. Lastly, because of homogenous Han Chinese ethnicity of our study sample, the findings may not generalize to other ethnic groups.

## Conclusions

In conclusion, our study presents population-based evidence that persons with migraine have a 20% higher risk of neovascular AMD compared to persons without migraine. Our study suggests that clinicians should be alert to the potential for neovascular AMD among migraine sufferers and should refer them for periodic fundus examinations by an ophthalmologist. Further studies are needed to confirm the association found in the present study in other regions and racial groups.
